# Practical Notes on Diagnosis and Treatment

**Published:** 1910-07-09

**Authors:** 


### Practical Notes on Diagnosis and Treatment.

**Urates.**

It is well to remember that this is the only deposit that occurs in urine (which has not decomposed) as the result of simple cooling, and hence if the urine was clear on voidance, a patient's mind can often be set at rest at once.—*Dr. F. J. Smith.*

**Rheumatoid Arthritis.**

You will do well to begin treatment in this disease by the local application of heat and by massage. But very often you cannot do this as people have to go about their business; then I believe the best thing is plenty of good food and iodide of iron.—*Dr. Hale White.*

**Uræmic Paralysis.**

While the occurrence of convulsions due to uræmia is a common experience, the existence of uræmic paralysis is much less frequent. Hemiplegia is the commonest form; it may be complete or involve only a single limb, or may be restricted to one side of the face.

**Intussusception.**

I should advise you not to make use of distension at all, but to open the abdomen as soon as a diagnosis of intussusception is made. If you do, however, decide to try distension, use air, and try it only in very early cases, certainly not later than twenty-four hours from the onset of symptoms.—*Mr. F. J. Steward.*

**Night-sweating.**

Before regarding night-sweats as a symptom of tuberculosis the following points must be considered: (1) The sweating must be worse in cold weather; (2) it must be on the forehead or round the neck; (3) it must not be due to heavy bedclothes, etc.; (4) if the sweating has existed all the child's life it is probable that the original cause was rickets.—*Dr. M. Williams.*

**Chicken Pox.**

Scarlatiniform rashes are not at all uncommon during the course of chicken pox, and are apt to lead to error in diagnosis. I have seen them both with and without pyrexia. There may be a more or less uniform flush, or there may be an actual punctate rash. There is no subsequent desquamation. The trunk is the most common situation, but occasionally the limbs are also involved; the marked injection of the fauces, which may be accompanied by an almost typical "scarlet" tongue, causes many cases to be notified as scarlatina.—*Dr. C. B. Ker.*

**Colchicum.**

This drug agrees best with younger patients and in first attacks; it is much less useful in the chronic gout of the older.—*Dr. Mitchell Bruce.*

**Post-nasal Catarrh.**

The swollen cervical glands which are associated with post-nasal irritation and catarrh may be favourably influenced by the application of iodine pigmentum to the naso-pharynx. This treatment will also do much good by putting an end to the flow of irritating matters into the stomach, and digestion will quickly recover, with general improvement all round.—*Dr. Eustace Smith.*

**Salicin in Psoriasis.**

Salicin has the advantage over arsenic and thyroid that it may be given in the spreading stage of psoriasis, and will often check it, while the other two are apt to increase the eruption. As far as this is concerned it has no contra-indications. While it is not always successful, it never seems to aggravate the disease, and the proportion of cures with it is higher than in the other two.—*Dr. Radcliffe Crocker.*

**The Menopause and Dyspepsia.**

One of the forms which the processes peculiar to the climacteric assume is an abnormal craving for sedatives. Alcohol is usually employed, and as a narcotic, not as a stimulant. This means that doses are taken large enough to act as an irritant on the gastric mucosa, which gives rise to symptoms of indigestion. There is a great deal of secret drinking at the time of the menopause, even among those who have been strictly temperate, so that the possibility of such a factor being at work in producing or maintaining a dyspepsia should not be forgotten.—*Dr. Leonard Williams.*

**Rectal Feeding.**

The administration of food in the form of nutrient suppositories is valueless and need not be considered. Rectal feeding is altogether overrated, and there are comparatively few cases in infants and children in which it is either necessary or advisable. A large amount of the benefit is due to the fluid rather than to the nutritive constituents of the injection. Complete rest to the stomach cannot be entirely obtained by this means, for the introduction of nutrient material into the lower bowel induces gastric secretion, pain, and vomiting. These symptoms may be sometimes relieved by giving a little milk and bicarbonate of soda by the mouth.—*Dr. Edmund Cautley.*

